# Platelet-Derived Growth Factors in Non-GIST Soft-Tissue Sarcomas Identify a Subgroup of Patients with Wide Resection Margins and Poor Disease-Specific Survival

**DOI:** 10.1155/2010/751304

**Published:** 2011-01-23

**Authors:** Thomas Karsten Kilvaer, Andrej Valkov, Sveinung W. Sorbye, Tom Donnem, Eivind Smeland, Roy Martin Bremnes, Lill-Tove Busund

**Affiliations:** ^1^Institute of Medical Biology, University of Tromsø, 9037 Tromsø, Norway; ^2^Department of Clinical Pathology, University Hospital of North Norway, 9038 Tromsø, Norway; ^3^Institute of Clinical Medicine, University of Tromsø, 9037 Tromsø, Norway; ^4^Department of Oncology, University Hospital of North Norway, 9038 Tromsø, Norway

## Abstract

*Background*. Optimal treatment of nongastrointestinal stromal tumor soft-tissue sarcomas (non-GIST STSs) is resection with wide margins. This study investigates the prognostic impact of the angiogenesis-associated platelet-derived growth factors (PDGFs) and their receptors (PDGFRs) in non-GIST STS patients with wide and nonwide resection margins. *Method*. Tumor samples and clinical data from 249 patients with non-GIST STS were obtained, and tissue microarrays were constructed for each specimen. Immunohistochemistry was used to evaluate the expression of PDGF-A, -B, -C, and -D and PDGFR-*α* and -*β*. *Results*. In the multivariate analysis of patients with wide resection margins, high expression of PDGF-B (*P* = .013, HR = 2.954, and 95% CI = 1.255–6.956) and the coexpression of PDGF-B and PDGFR-*α* (overall; *P* = .016, high-low/low-high; *P* = .051, HR = 2.678, 95% CI = 0.996–7.200, high/high; *P* = .004, HR = 3.930, 95% CI = 1.542–10.015) were independent negative prognostic markers for disease-specific survival. *Conclusion.* PDGF-B and the coexpression of PDGF-B and PDGFR-*α* are strong and independent prognostic factors in non-GIST STSs with wide resection margins.

## 1. Introduction

Soft-tissue sarcomas (STSs) are mesenchymal-derived tumors comprising about 0.5% of the annual cancer incidence with an estimate of ten thousand new patients and nearly four thousand related deaths in the USA in 2009 [1]. The STS group consists of more than 50 histological entities [2]. Because of the low incidence and similar ancestry of these tumors it is convenient to group them together when conducting studies [2, 3]. A proposed way to group these tumors is Ewing family tumors, gastrointestinal stromal tumors (GISTs), and non-GIST STSs, with the latter group consisting of the remaining tumors [3]. Despite improvements in therapy over the last decades the disease-specific survival (DSS) and progression-free survival (PFS) of sarcoma patients are still poor. The main treatment is resection with wide margins, while radiotherapy is often used in high-grade tumors with both marginal and wide resection margins [4, 5]. Even when wide resection margins are obtained, a relatively high proportion of patients die [6, 7]. Several adjuvant chemotherapy-regimes are used for the treatment of sarcomas, but with the exception of childhood rhabdomyosarcomas, Ewing family tumors and GISTs, studies are inconclusive on the effects of these agents [4, 8, 9]. Identification of the subgroup of patients, with wide resection margins and low survival, could prove important, as these patients might benefit from adjuvant therapy.

The platelet-derived growth factor (PDGF) group of signaling molecules consists of four proteins forming five possible ligands *in vivo*, namely PDGF-AA, PDGF-AB, PDGF-BB, PDGF-CC, and PDGF-DD [10]. The platelet-derived growth factor receptors (PDGFRs) are structurally related tyrosine-kinase receptors consisting of either *α*- or *β*-chains forming three possible receptors: PDGFR-*αα*, PDGFR-*αβ*, and PDGFR-*ββ*. The PDGF-AA binds exclusively to the PDGFR-*αα*, while PDGF-AB and -CC bind to both the PDGFR-*αα* and PDGFR-*αβ*; PDGF-DD binds both PDGFR-*αβ* and PDGFR-*ββ*, while PDGF-BB binds all PDGFRs [10].

PDGFs and PDGFRs play a major role in angiogenesis, the recruitment and regulation of tumor stroma, and the regulation of tumor interstitial fluid pressure (IFP) [11]. In addition PDGFs have been shown to function as powerful transforming growth factors, leading cells to progress through cell-cycle and avoid apoptosis [12].

The PDGF/PDGFR pathways have previously been implicated in several sarcomas including GIST, dermatofibrosarcoma protuberans, childhood rhabdomyosarcoma, Kaposis sarcoma, osteosarcoma, and Ewing family sarcoma [11, 13–19]. Regarding non-GIST STSs, there are no conclusive studies on the prognostic impact of PDGF/PDGFRs. Knowledge about the expression and prognostic impact of PDGFs and PDGFRs may be important in identifying patients with wide resection margins and low DSS. With the emerging class of selective small molecule inhibitors targeting these pathways, this might be particularly important. The study presented herein investigates the prognostic impact of PDGFs and PDGFRs in non-GIST STS with wide and nonwide resection margins.

## 2. Patients and Methods

### 2.1. Patients and Clinical Samples

Primary tumor tissue from anonymized patients diagnosed with non-GIST STS at the University Hospital of North Norway and the hospitals of Arkhangelsk county, Russia, from 1973 through 2006, were collected. In total, 496 patients were registered from the hospital databases. Of these 247 patients were excluded from the study because of missing clinical data (*n* = 86) or inadequate paraffin-embedded fixed tissue blocks (*n* = 161). Thus, 249 patients with complete medical records and adequate paraffin-embedded tissue blocks were eligible.

This report includes followup data as of September 2009. The median followup was 37.6 (range 0.1–391.7) months. Complete demographic and clinical data were collected retrospectively. Formalin-fixed and paraffin-embedded tumor specimens were obtained from the archives of the Departments of Pathology at the University Hospital of North Norway and the hospitals of Arkhangelsk county, Russia. The tumors were graded according to the French Fédération Nationale des Centres de Lutte Contre le Cancer (FNCLCC) system and histologically subtyped according to the World Health Organization guidelines [2, 20]. Wide resection margins were defined as wide local resection with free microscopic margins or amputation of the affected limb or organ. Nonwide resection margins were defined as marginal or intralesional resection margins, or no surgery. 

### 2.2. Microarray Construction

All sarcomas were histologically reviewed by two trained pathologists (S. Sorbye and A. Valkov), and the most representative areas of tumor cells (neoplastic mesenchymal cells) were carefully selected and marked on the hematoxylin and eosin (H/E) slide and sampled for the tissue microarray (TMA) blocks. The TMAs were assembled using a tissue-arraying instrument (Beecher Instruments, Silver Springs, MD, USA). The detailed methodology has been previously reported in [21]. Briefly, we used a 0.6 mm diameter stylet, and the study specimens were routinely sampled with four replicate core samples from different areas of neoplastic tissue. Normal tissue from the patients was used as staining control.

To include all core samples, 12 TMA blocks were constructed. Multiple 5-*μ*m sections were cut with a Micron microtome (HM355S) and stained by specific antibodies for immunohistochemistry (IHC) analysis. 

### 2.3. Immunohistochemistry

The applied antibodies were subjected to in-house validation by the manufacturer for IHC analysis on paraffin-embedded material. The antibodies used in the study were as follows: PDGF-AA (goat polyclonal; AB-221-NA; R&D Systems; 1 : 200), PDGF-AB/BB (rabbit polyclonal; RB-9257; Neomarkers; 1 : 15), PDGF-CC (goat polyclonal; GT15151; Neuromics; 1 : 80), PDGF-DD (goat polyclonal; AF1159; R&D Systems; 1 : 400), PDGFR-*α* (rabbit polyclonal; RB-9027; Neomarkers; 1 : 75), and PDGFR-*β* (rabbit polyclonal; RB-9032; Neomarkers; 1 : 25). 

Sections were deparaffinized with xylene and rehydrated with ethanol. Antigen retrieval of PDGF-A, -B, -C, and -D was performed by placing the specimen in 0.01 M citrate buffer at pH 6.0 and exposing them to repeated (×2) microwave heating of 10 min at 450 W. PDGF-A, -B, and -C were stained using peroxydase/DAB (Dako EnVision+System-HRP/DAB). The primary antibodies were incubated for 30 min in room temperature. PDGF-D was visualized by adding a secondary antibody conjugated with Biotin, followed by an Avidin/Biotin/Peroxydase complex (Vectastein ABC Elite kit from Vector Laboratories). The primary antibody was incubated overnight at 4°C. Finally, all slides were counterstained with hematoxylin to visualize the nuclei. 

The receptors (PDGFR-*α* and -*β*) were stained using Ventana BenchMark XT (Ventana Medical Systems Inc.), procedure iView DAB. Antigen retrieval was done in Tris/EDTA buffer at pH 8.4 for 30 min (PDGFR-*α*) or 60 min (PDGFR-*β*) at 37°C. The primary antibodies were incubated for 30 min in room temperature.

For each antibody, included negative staining controls, all TMA stainings were done in a single experiment. 

### 2.4. Scoring of Immunohistochemistry

The ARIOL imaging system (Genetix, San Jose, CA) was used to scan the slides of antibody staining of the TMAs. The slides were loaded in the automated slide loader (Applied Imaging SL 50), and the specimens were scanned at low resolution (1.25×) and high resolution (20x) using the Olympus BX 61 microscope with an automated platform (Prior). Representative and viable tissue sections were scored manually on computer screen semiquantitatively for cytoplasmic staining. The dominant staining intensity was scored as 0: negative, 1: weak, 2: intermediate, and 3: strong. All samples were anonymized and independently scored by two trained pathologists (A. Valkov and S. Sorbye). When assessing a variable for a given core, the observers were blinded to the scores of the other variables and to the outcome. Mean score for duplicate cores from each individual was calculated separately.

**Figure 1:**
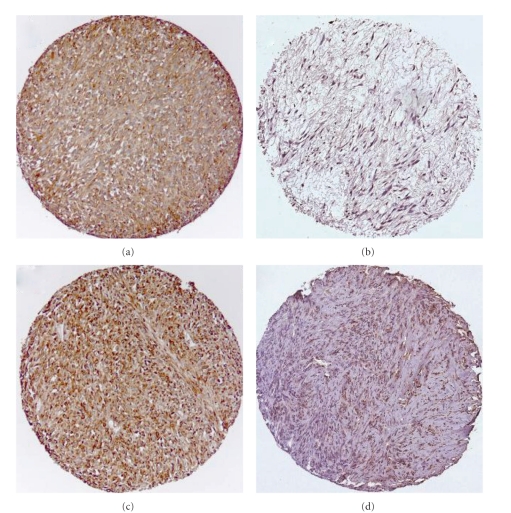
IHC analysis of TMA of nongastrointestinal stromal tumor soft-tissue sarcoma representing different scores for tumor cell PDGF-B and PDGFR-*α*. (a) Tumor cell PDGF-B high score; (b) tumor cell PDGF-B low score; (c) tumor cell PDGFR-*α* high score; (d) tumor cell PDGFR-*α* low score. Abbreviations: IHC: immunohistochemistry; TMA: tissue microarray; PDGF: platelet-derived growth factor; PDGFR: platelet-derived growth factor receptor.

High expression in tumor cells was defined as score ≥1.5 (PDGF-A, PDGF-C, and PDGF-B) and ≥2 (PDGF-D, PDGFR-*α*, and PDGFR-*β*) (Figure 1). 

### 2.5. Statistical Methods

All statistical analyses were done using the statistical package SPSS (Chicago, IL, USA), version 16. The IHC scores from each observer were compared for interobserver reliability by use of a two-way random effect model with absolute agreement definition. The intraclass correlation coefficient (reliability coefficient) was obtained from these results. The Chi-square test and Fishers Exact test were used to examine the association between molecular marker expression and various clinicopathological parameters. Univariate analyses were done using the Kaplan-Meier method, and statistical significance between survival curves was assessed by the log-rank test. DSS was determined from the date of diagnosis to the time of cancer-related death. To assess the independent value of different pretreatment variables on survival, in the presence of other variables, multivariate analyses were carried out using the Cox proportional hazards model. Only variables of significant value from the univariate analyses were entered into the Cox regression analyses. Probability for stepwise entry and removal was set at.05 and.10, respectively. The significance level used for all statistical tests was *P* < .05. 

### 2.6. Ethical Clearance

The National Data Inspection Board and The Regional Committee for Research Ethics approved the study.

## 3. Results

### 3.1. Clinopathological Variables

The clinopathological variables are summarized in Table 1. The median age was 59 (range 0–91) years, 56% were female; 167 patients were Norwegian and 82 Russian. The Non-GIST STSs comprised 249 tumors including angiosarcoma (*n* = 13), fibrosarcoma (*n* = 20), leiomyosarcoma (*n* = 64), liposarcoma (*n* = 34), pleomorphic sarcoma (*n* = 58), neurofibrosarcoma/malignant peripheral nerve sheath tumor (MPNST, *n* = 11), rhabdomyosarcoma (*n* = 16), synovial sarcoma (*n* = 16), and unspecified sarcoma (*n* = 17). The tumor origins were distributed as follows: 36% extremities, 19% trunk, 15% retroperitoneal, 7% head/neck, and 23% visceral. Of 228 patients who underwent surgery, 53% received surgery alone, 24% surgery and radiotherapy, 18% surgery and chemotherapy, and 6% surgery, radiotherapy, and chemotherapy. Besides, 21 patients did not undergo surgery due to inoperable tumor (*n* = 11), high age/other serious diseases (*n* = 5), STS confirmed at autopsy (*n* = 3) and patient refusal (*n* = 2). Of these unresected patients, seven patients received chemotherapy and/or radiotherapy, whereas 14 patients received no anticancer therapy. 

### 3.2. Interobserver Variability

Interobserver scoring agreement was tested for one ligand (PDGF-B) and one receptor (PDFGR-*α*). The intraclass correlation coefficients were 0.890 for PDGF-B (*P* < .001) and 0.892 for PDFGR-*α* (*P* < .001) indicating good reproducibility between the two investigating pathologists. 

### 3.3. Expression of PDGFs/PDGFRs and Their Correlations

PDGF/PDGFR-expression was observed in the cytoplasm of tumor cells. No correlation was seen between increased tumor PDGF/PDGFR expression and tumor depth, surgery, radiotherapy, or chemotherapy. Among the most interesting correlations, PDGFR-*α* correlated with malignancy grade (high expression; grade 1: 25%, grade 2: 39%, grade 3: 54%, *P* = .003) and PDGFR-*β* was more often expressed in patients with metastasis at diagnosis (35%, versus no metastasis at diagnosis, 18%, *P* = .015). 

### 3.4. Univariate Analyses

Among demographic and clinicopathological variables in the total material, patient nationality (*P* = .011), histological entity (*P* = .001), tumor size (*P* = .027), malignancy grade (*P* < .001), tumor depth (*P* < .001), metastasis at diagnosis (*P* < .001), surgery (*P* < .001), and resection margins (*P* < .001) were significant prognostic indicators of DSS (Table 1). 

In the subgroup with wide resection margins, patient nationality (*P* < .001), malignancy grade (*P* < .001), tumor depth (*P* = .009), and metastasis at diagnosis (*P* < .001) were significant prognostic indicators of DSS. In the subgroup with nonwide resection margins, malignancy grade (*P* < .001), metastasis at diagnosis (*P* < .001), surgery (*P* < .001), and histological entity (*P* = .004) were significant prognostic indicators of DSS.

**Figure 2:**
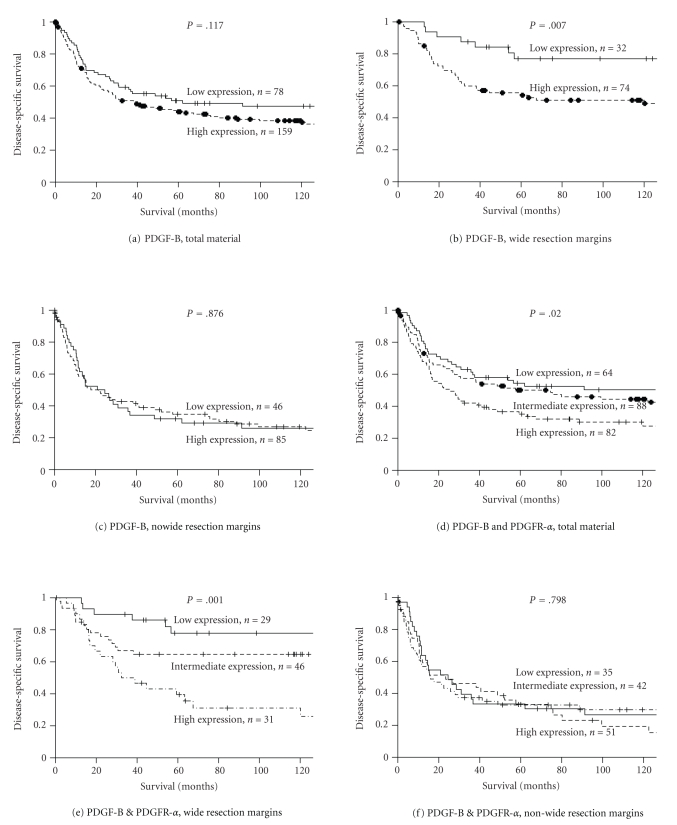
Disease-specific survival curves for PDGF-B and the coexpression of PDGF-B & PDGFR-*α* in the overall material and in patients with wide and nonwide resection margins. (a) PDGF-B, total material; (b) PDGF-B, wide resection margins; (c) PDGF-B, nonwide resection margins; (d) coexpression of PDGF-B and PDGFR-*α*, total material; (e) coexpression of PDGF-B and PDGFR-*α*, wide resection margins; (f) coexpression of PDGF-B and PDGFR-*α*, nonwide resection margins. Abbreviations: PDGF: platelet-derived growth factor; PDGFR: platelet-derived growth factor receptor.

The influence on DSS by the PDGFs and PDGFRs is shown in Table 2 and Figure 2. In the total material, high expressions of PDGFR-*α* (*P* = .004) and PDGFR-*β* (*P* = .047) were significant negative prognostic indicators of DSS. In the subgroup with wide resection margins, high expressions of PDGF-B (*P* = .007), PDGF-D (*P* = .029), PDGFR-*α* (*P* = .001), and PDGFR-*β* (*P* = .022) were significant negative prognostic indicators of DSS, while in the subgroup with nonwide resection margins none of the PDGFs or PDGFRs were significant indicators of DSS. 

### 3.5. Multivariate Cox Proportional Hazards Analyses

Results of the multivariate analyses are presented in Tables 3 and 4. In the total material, tumor depth (*P* = .019), tumor size (*P* = .034), high malignancy grade (*P* < .001), lack of surgery (*P* < .001), nonwide resection margins (*P* = .013), and metastasis at diagnosis (*P* < .001) were significant independent prognostic indicators of DSS (Table 3). In the subgroup with wide resection margins, Russian nationality (*P* = .012), high malignancy grade (*P* = .005), metastasis at diagnosis (*P* = .001), and high expression of PDGF-B (*P* = .013, HR = 2.954, 95% CI = 1.255–6.956) were significant independent prognostic indicators of DSS (Table 4). In the subgroup with nonwide resection margins, high malignancy grade (*P* < .001), lack of surgery (*P* < .001), and metastasis at diagnosis (*P* < .001) were significant independent prognostic indicators of DSS. 

### 3.6. Coexpression of PDGF-B and PDGFR-*α*

It is pertinent to assess the coexpression between PDGFs and their receptors as these can represent additive and/or synergic effects on DSS. In univariate analyses, the coexpression of PDGF-B and PDGFR-*α* was a significant negative prognostic indicator of DSS both in the total material (*P* = .020) and in the subgroup with wide resection margins (*P* = .001). In the latter group, the coexpression was a significant independent negative prognostic indicator of DSS (overall; *P* = .016, intermediate; *P* = .051, HR = 2.678, 95% CI = 0.996–7.200, high; *P* = .004, HR = 3.930, 95% CI = 1.542–10.015).

## 4. Discussion

The main treatment for non-GIST STSs is surgery with wide resection margins [4]. The 5-year survival is 30% in the group with nonwide resection margins and 60% among those with wide resection margins. The explanation for the modest survival, despite wide resection margins, might be micrometastasis into the surrounding tissue, lymphogenic regional spread, or hematogenous metastasis. Any possibility to identify those patients who will subsequently succumb to progression and metastasis from their resected sarcoma within the wide resection margin group will be pivotal, as these patients may benefit from adjuvant therapy.

We present a large-scale retrospective study of the prognostic impact of PDGF-A, -B, -C, and -D and PDGFR-*α* and -*β* in non-GIST STS patients. High expression of PDGF-B and the coexpression of PDGF-B and PDGFR-*α* were significant independent negative prognostic indicators of DSS in those with wide resection margins. To our knowledge this is the first evaluation of PDGFs and PDGFRs in non-GIST STSs according to resection margins [22]. 

The major limitation of this study consists of tumor heterogeneity as different types of non-GIST STS, diverse disease sites, as well as different therapies are included. These points are, at least partly, accounted for by the multivariate analysis. Recent studies have suggested new ways of grouping STSs according to their mutational status in addition to histology [22]. A homologous population with knowledge of mutational status of common sarcoma mutations would have been interesting, but this is difficult to arrange in the setting of a retrospective study.

 The major strengths of this study lie in the size of the cohort, the consistency of the clinical variables with previous studies on sarcomas, and the biological soundness of our findings.

In 1994, Wang et al. reported a positive correlation between increased PDGF-B expression and high histological malignancy grade of STSs using a set of 56 STSs of all grades including benign tumors [23]. Herein, we did not observe a correlation between PDGF-B expression and histological grade, but PDGF-B appeared to be a significant independent negative prognostic marker for DSS in patients with wide resection margins. 

Experiments in mice have shown involvement of endothelial-derived PDGF-B, in both pericyte and vascular smooth muscle cell (VSM) recruitment and stabilization, during blood vessel formation [24–26]. Pericytes have been implicated in differentiation and stabilization of blood vessels, and VSMs are an important component of blood vessel walls. As adequate blood supply is pivotal in tumor formation, tumor-derived PDGF-B could represent a way for recruitment of pericytes and VSMs, leading to tumor growth and increased viability and tumor forming capabilities of metastatic tumors. High tumor interstitial fluid pressure (IFP) has been shown to lower chemotherapeutic response due to lower transcapillary transport of chemotherapeutic agents [27]. PDGF-B has been shown to increase IFP and may play a role in tumors showing unexpected poor response to chemotherapy [11]. Studies in mice demonstrated that blocking PDGFs and PDGFRs resulted in better response to chemotherapy in several experimental tumor types [28, 29]. These findings indicate that increased expression of PDGFs and PDGFRs might perturb the effect of chemotherapy and therefore contribute to explain why the response to chemotherapy remains controversial in non-GIST STSs. PDGF-B primarily signals through PDGFR-*β* but has also been shown to signal through PDGFR-*α* [10]. Upon receptor activation, PDGF-B has strong transforming capabilities, activating several intracellular pathways, including PI3K, PLC*γ*, SRC, and RAS. Activation of these pathways might lead to increased cell cycling and avoidance of apoptosis [12]. In univariate analysis, high expression of PDGF-D was a significant negative prognostic marker in non-GIST STSs with wide resection margins. PDGF-D has previously been shown to exhibit extensive transforming and angiogenic abilities [30]. Vessels formed in PDGF-D-driven tumors show great similarity to vessels formed by PDGF-B-driven tumors, suggesting that PDGF-D, in absence of PDGF-B, can take over some or all of PDGF-Bs angiogenic functions [30]. Further, both PDGFR-*α* and -*β* were significant negative prognostic markers for non-GIST STSs with wide resection margins. PDGFRs have been regarded as a “driving force” in many human cancers, including GISTs, through autoactivation and constitutive signaling [19]. The fact that PDGFR-*α* correlated with malignancy grade and that PDGFR-*β* correlated with metastasis at diagnosis suggests that this might also be the case in non-GIST STSs, although further investigation is warranted.

We also found the coexpression of PDGF-B and PDGFR-*α* to have a significant independent negative prognostic impact in non-GIST STS with wide resection margins. A significantly lower survival in the high-high group versus the low-low and the high-low/low-high groups suggests an additive or possibly synergic effect (Figure 2). The exact mechanism for this finding is not clear, but there are several possible explanations. As previously mentioned, PDGF-B preferably signals through PDGFR-*β* but can also signal through PDGFR-*α*. When both PDGFRs and PDGF-B are expressed in the tumor it is likely that both pathways are simultaneously stimulated by PDGF-B or by consecutive stimulation of the PDGFR-*β* with constitutively active PDGFR-*α*. Part of the effect might also come from PDGF-B interactions with stromal components like pericytes and VSMs. Simultaneous stimulation of both PDGFR axes, together with the proposed angiogenic and stromal regulatory effects of PDGF-B, may explain the decreased DSS in non-GIST STS patients with wide resection margins.

Small-molecule inhibitors of tyrosine kinase receptors have been used in the treatment of GISTs, dermatofibrosarcoma protuberans, chronic myelogenous leukemia, and others, demonstrating great therapeutic potential [31]. Cell line experiments and animal studies suggest synergistic effects and reduced side effects of these inhibitory agents together with conventional chemo- or radiotherapy [28, 29]. 

## 5. Conclusion

Despite wide resection margins, one third of patients still die of non-GIST STS. We have identified high expression of PDGF-B to be an independent negative prognostic factor for DSS in non-GIST STS patients with wide resection margins. Further, PDGF-B and PDGFR-*α* coexpression revealed a wide resection margin subgroup with particularly poor DSS. Our results indicate involvement of PDGF and/or PDGFRs in non-GIST STS pathogenesis. The mechanisms responsible for this involvement have to be further elucidated and finally validated in prospective clinical trials. With the evolving small-molecule inhibitors targeting these pathways, PDGFs and PDGFRs may become important targets in the treatment of non-GIST STSs. 

##  Funding

This study was funded by the Northern Norway Health Authority, The Norwegian Childhood Cancer Network, The Norwegian Sarcoma Group, and The Norwegian Cancer Society. The funding sources had no influence on the study design, data collection, analysis, and interpretation of data, in the writing of the paper, or in the decision to submit the paper for publication. 

##  Conflict of Interests

The authors declare that they have no conflict of interests. 

## Figures and Tables

**Table 1:** Prognostic clinicopathological variables as predictors for disease-specific survival in 249 nongastrointestinal stromal tumor soft-tissue sarcomas (univariate analyses; log-rank test).

Characteristics	Patients (*n*)	Patients (%)	Median survival (months)	5-Year survival (%)	*P*
Age					
≤20 years	20	8	15	40	.126
21–60 years	113	45	68	52	
>60 years	116	47	30	40	
Gender					
Male	110	44	41	46	.390
Female	139	56	45	45	
Patient nationality					
Norwegian	167	67	63	51	.011
Russian	82	33	22	34	
Histological entity					
Pleomorphic sarcoma	58	23	54	47	.001
Leiomyosarcoma	64	26	48	48
Liposarcoma	34	14	NR	67
Fibrosarcoma	20	8	44	50
Angiosarcoma	13	5	10	31
Rhabdomyosarcoma	16	6	17	38
MPNST	11	4	49	45
Synovial sarcoma	16	6	31	29
Sarcoma NOS	17	7	9	18
Tumor localization					
Extremities	89	36	100	53	.348
Trunk	47	29	32	44
Retroperitoneum	37	25	25	38
Head/neck	18	7	15	41
Visceral	58	23	30	42
Tumor size					
≤5 cm	74	30	127	57	.027
5–10 cm	91	37	44	45
>10 cm	81	32	28	37
Missing	3	1		
Malignancy grade					
1	61	25	NR	74	<.001
2	98	39	41	45	
3	90	36	16	26	
Tumor depth					
Superficial	17	7	NR	93	<.001
Deep	232	93	36	42	
Metastasis at diagnosis					
No	206	83	76	53	<.001
Yes	43	17	10	10	
Surgery					
Yes	228	91	59	50	<.001
No	21	9	4	0	
Resection margins					
Wide	108	43	NR	62	<.001
Nonwide/no surgery	141	57	21	33	
Chemotherapy					
No	191	77	52	47	.424
Yes	58	23	29	40	
Radiotherapy					
No	176	71	48	46	.590
Yes	73	29	38	43	

Abbreviations: NR: not reached; MPNST: malignant peripheral nerve sheath tumor; NOS: not otherwise specified.

**Table 2:** Tumor expression of PDGFs and PDGFRs and their prediction for disease-specific survival in patients with nongastrointestinal stromal tumor soft-tissue sarcoma in the total material (univariate analyses; log-rank test, *n* = 249) and in subgroups with wide and nonwide resection margins (univariate analyses; log-rank test, *n* = 108 and 141, resp.).

Marker expression	Total material					Wide resection margins					Nonwide resection margins				
	Patients (*n*)	Patients (%)	Median survival (months)	5-Year survival (%)	*P*	Patients (*n*)	Patients (%)	Median survival (months)	5-Year survival (%)	*P*	Patients (*n*)	Patients (%)	Median survival (months)	5-Year survival (%)	*P*
PDGF-A															
Low	99	40	52	47	.253	47	44	NR	65	.190	52	37	18	29	.985
High	138	55	41	46		60	56	127	59		78	56	23	36	
Missing	12	5				1	1				11	8			

PDGF-B															
Low	78	31	62	51	.117	32	30	NR	77	.007	46	33	25	32	.876
High	159	64	38	44		74	69	120	54		85	60	21	35	
Missing	12	5				2	2				10	7			

PDGF-C															
Low	63	25	57	48	.226	25	23	NR	70	.148	38	27	26	33	.434
High	173	70	37	45		78	72	NR	59		95	67	15	32	
Missing	13	5				5	5				8	6			

PDGF-D															
Low	158	63	75	51	.095	78	72	NR	67	.029	80	57	21	34	.606
High	81	33	31	37		27	25	39	46		54	38	15	33	
Missing	10	4				3	3				7	5			

PDGFR-*α*															
Low	139	56	91	54	.004	73	68	NR	71	.001	66	47	25	35	.924
High	96	39	27	35		34	32	39	42		62	44	15	32	
Missing	14	6				1	1				13	9			

PDGFR-*β*															
Low	183	74	63	51	.047	88	82	NR	67	.022	95	67	23	35	.781
High	48	19	22	33		17	16	31	35		31	22	11	32	
Missing	18	7				3	3				15	11			

PDGF-B & PDGFR-*α*															
Low	64	26	NR	54	.020	29	27	NR	78	.001	35	25	25	33	.798
Intermediate	88	35	75	50		46	43	NR	65		42	30	23	33	
High	82	33	26	35		31	29	32	40		51	36	16	33	
Missing	15	6				2	2				13	9			

Abbreviations: PDGF: platelet-derived growth factor; PDGFR: platelet-derived growth factor receptor; NR: not reached.

**Table 3:** Result of the Cox regression analysis among all patients.

Factor	Hazard ratio	95% CI	*P*
Tumor depth			
Superficial	1.000		
Deep	9.765	1.339–71.189	.025

Tumor size			.028*
≤5 cm	1.000		
5–10 cm	1.379	0.855–2.223	.187
>10 cm	1.960	1.192–3.221	.008

Malignancy grade			<.001*
1	1.000		
2	2.914	1.605–5.292	<.001
3	4.600	2.527–8.373	<.001

Surgery			
Yes	1.000		
No	8.628	4.269–17.438	<.001

Resection margins			
Wide	1.000		
Nonwide	1.847	1.243–2.744	.003

Metastasis at time of diagnosis			
No	1.000		
Yes	2.562	1.644–3.991	<.001

*Overall significance as a prognostic factor.

**Table 4:** Results of the Cox regression analysis among patients with wide resection margins.

Factor	Hazard ratio	95% CI	*P*
Patient nationality			
Norwegian	1.000		
Russian	2.292	1.199–4.383	.012

Malignancy grade			.005*
1	1.000		
2	4.438	1.262–15.609	.020
3	7.368	2.138–25.389	.002

Metastasis at time of diagnosis			
No	1.000		
Yes	3.939	1.801–8.613	.001

PDGF-B			
Low	1.000		
High	2.954	1.255–6.956	.013

*Overall significance as a prognostic factor. Abbreviations: PDGF: platelet-derived growth factor.
